# Modes of Transmission of Influenza B Virus in Households

**DOI:** 10.1371/journal.pone.0108850

**Published:** 2014-09-30

**Authors:** Benjamin J. Cowling, Dennis K. M. Ip, Vicky J. Fang, Piyarat Suntarattiwong, Sonja J. Olsen, Jens Levy, Timothy M. Uyeki, Gabriel M. Leung, J. S. Malik Peiris, Tawee Chotpitayasunondh, Hiroshi Nishiura, J. Mark Simmerman

**Affiliations:** 1 School of Public Health, Li Ka Shing Faculty of Medicine, The University of Hong Kong, Hong Kong Special Administrative Region, China; 2 Queen Sirikit National Institute of Child Health, Bangkok, Thailand; 3 Influenza Program, Thailand MOPH-US CDC Collaboration, Nonthaburi, Thailand; 4 Influenza Division, US Centers for Disease Control and Prevention, Atlanta, Georgia, United States of America; 5 Centre for Influenza Research, Li Ka Shing Faculty of Medicine, The University of Hong Kong, Hong Kong Special Administrative Region, China; 6 Graduate School of Medicine, The University of Tokyo, Bunkyo-ku, Tokyo, Japan; 7 Epidemiology and Medical Affairs, Sanofi Pasteur, Bangkok, Thailand; Arizona State University, United States of America

## Abstract

**Introduction:**

While influenza A and B viruses can be transmitted via respiratory droplets, the importance of small droplet nuclei “aerosols” in transmission is controversial.

**Methods and Findings:**

In Hong Kong and Bangkok, in 2008–11, subjects were recruited from outpatient clinics if they had recent onset of acute respiratory illness and none of their household contacts were ill. Following a positive rapid influenza diagnostic test result, subjects were randomly allocated to one of three household-based interventions: hand hygiene, hand hygiene plus face masks, and a control group. Index cases plus their household contacts were followed for 7–10 days to identify secondary infections by reverse transcription polymerase chain reaction (RT-PCR) testing of respiratory specimens. Index cases with RT-PCR-confirmed influenza B were included in the present analyses. We used a mathematical model to make inferences on the modes of transmission, facilitated by apparent differences in clinical presentation of secondary infections resulting from aerosol transmission. We estimated that approximately 37% and 26% of influenza B virus transmission was via the aerosol mode in households in Hong Kong and Bangkok, respectively. In the fitted model, influenza B virus infections were associated with a 56%–72% risk of fever plus cough if infected via aerosol route, and a 23%–31% risk of fever plus cough if infected via the other two modes of transmission.

**Conclusions:**

Aerosol transmission may be an important mode of spread of influenza B virus. The point estimates of aerosol transmission were slightly lower for influenza B virus compared to previously published estimates for influenza A virus in both Hong Kong and Bangkok. Caution should be taken in interpreting these findings because of the multiple assumptions inherent in the model, including that there is limited biological evidence to date supporting a difference in the clinical features of influenza B virus infection by different modes.

## Introduction

Influenza viruses are believed to be spread between humans through a number of modes of transmission, including primarily through inhalation of respiratory droplets containing infectious virus, and possible contact of respiratory secretions containing infectious virus with mucous membranes. A distinction is sometimes drawn between larger versus smaller respiratory droplets, as large droplets quickly fall to the ground [1], [2], while droplet nuclei can remain suspended in the air for prolonged periods because of their low settling velocity [3]. However, aerosols are easily removed from the environment through ventilation, and infectious virus suspended in aerosols could be fragile and easily lose infectivity. The threshold for small particles is typically drawn in the range 5 µm to 20 µm [3]–[5]. Only a small number of pathogens are thought to transmit via aerosols, including varicella virus, *M. tuberculosis* and rubeola virus (measles) [6]. The potential for influenza virus to spread by aerosols remains controversial [3]–[5], [7], [8]. There is growing evidence that influenza A virus can spread by aerosols [3]–[5], [8]–[10], but less discussion over the potential role of aerosols in influenza B virus transmission with limited published literature. Infectious influenza B virus can be detected in the aerosol fraction (particles <5 µm) of exhaled breath of subjects with influenza B virus infection [11].

Influenza B viruses can infect all age groups. Compared to influenza A viruses, infections with influenza B virus are more commonly identified in children compared to adults [12], perhaps because of slower evolutionary rates [13] leading to greater herd immunity among adults. Influenza B virus infections can cause severe illness in all ages [14], and the mortality impact of influenza B epidemics in populations is generally estimated to be comparable to the impact of influenza A(H1N1) epidemics but somewhat less than influenza A(H3N2) epidemics, with the majority of excess deaths occurring in the very young and very old [15]–[18].

Historical volunteer challenge studies reported a difference in clinical presentation of influenza A virus infections depending on the mode of infection [9]. In one classic study, 23 people were experimentally inoculated with aerosols, 7 subsequently had serologic evidence of infection and virus was recovered from one additional volunteer without serologic evidence of infection, and 4 of those 8 had typical ILI with fever [19].

In another study, 24 people were inoculated intranasally and had milder illness than people with naturally-acquired illness [20]. In some infectious diseases (e.g. smallpox, plague), the clinical severity is known to depend on the mode of acquisition, and this property has recently been termed ‘anisotropic’ infection [21]. We previously assumed that influenza A virus also has the anisotropic property, and based on that property, further assuming that hand hygiene and face masks act primarily against contact and large droplet transmission respectively, we estimated that up to 50% of influenza A virus transmission within households in Hong Kong and Bangkok occur via the aerosol route [9]. Here, we propose that the same anisotropic nature may hold for influenza B virus infections, specifically that the mode of exposure leading to an infection may affect the pattern in subsequent signs and symptoms [21], and we use the same modeling framework to infer the proportion of household transmission of influenza B virus that occurs via the aerosol route.

## Methods

### Sources of Data

During 2008–2011, large randomized controlled trials were conducted in Hong Kong and Bangkok to study the efficacy of hand hygiene and surgical face masks in reducing influenza virus transmission in households [22], [23]. In each study, local residents who had acute respiratory illness and living in a household with at least 2 other people of whom none had reported acute respiratory illness in the preceding 14 days were enrolled. Pooled nasal and throat swab (NTS) specimens were collected from each participant for testing with the QuickVue Influenza A+B rapid diagnostic test (Quidel, San Diego, California). Participants with a positive rapid influenza test result were further followed up along with their household contacts. Households were randomly allocated in equal proportions into one of three intervention groups: (1) a control intervention, (2) control plus hand hygiene intervention, and (3) control plus facemasks and hand hygiene interventions. A home visit was scheduled as soon as possible after randomization to implement the intervention, collect baseline demographic data and NTS specimens from all household contacts aged ≥2 years, and to describe the information to be recorded in daily symptom diaries. Further home visits were scheduled at 3 and 6 days after the first home visit to monitor adherence to intervention and to collect further NTS specimens from all household contacts regardless of illness. The two study protocols were very similar, and notable differences are summarized in Table 1.

**Table 1:** Minor differences between the study designs in Hong Kong and Bangkok.

Study component	Hong Kong	Bangkok
Recruitment locations	45 public and private outpatient clinics across Hong Kong(population 7 million).	Outpatient department of a large pediatric public hospitalin Bangkok (population 8 million).
Study period	January 2008–June 2009	April 2008–February 2011
Age of index cases	Any age	Children 1 m to 15 y of age
Eligibility of index case (symptoms)	Presenting with at least two of: fever ≥37.8°C, cough,sore throat, headache, runny nose, phlegm, and myalgia; living withat least two other people.	For <2 years: fever >38°C *and* one or more of the followingsymptoms; nasal congestion, cough, conjunctivitis, respiratorydistress, sore throat, new seizure. For >2 years:Presenting with influenza-like illness (fever plus cough or sore throat);living with at least two other people.
Exclusion criteria	Recent (within 14 d) acute respiratoryillness in anyhousehold member	Recent (within 7 d) influenza-like illness in any household member;recent (within 12 m) influenza vaccination in any household member.
Hand hygiene intervention	Distribution of alcohol hand rub to each household memberin addition to liquid hand soapto the household	Distribution of liquid hand soap to the household
Measurement of body temperature	All households were provided and instructed in the useof a free tympanic thermometer andasked to record their bodytemperature daily.	Thermometers were not provided to households,and participants recorded either measured bodytemperature or ‘feverishness’.

All NTS specimens were tested by reverse-transcription polymerase chain reaction (RT-PCR) for influenza A and B viruses using standard methods as described elsewhere [22], [23]. In the present analyses only the households of index cases with RT-PCR-confirmed influenza B virus infection are included; results for index cases with influenza A were reported elsewhere [9].

In the present analyses, we used data on influenza B virus transmission in families from the studies in Hong Kong and Bangkok. Specifically, we identified all index cases with confirmed influenza B virus infection, and their household contacts. We then determined which household contacts had RT-PCR confirmed infection, the corresponding times of illness onset, and whether fever and cough were reported. In the analyses we also used the allocated intervention group for each household, and the age of each household contact.

### Ethics Statement

All subjects 18 years of age and older gave written informed consent, and proxy written consent was obtained from parents or legal guardians for children aged 17 years old or younger. The protocols for the studies in Hong Kong and in Bangkok were approved by Institutional Review Board of the University of Hong Kong, and the Institutional Review Board of Queen Sirikit Hospital Bangkok, respectively [22], [23].

### Statistical Analysis

We used the Nelson-Aalen non-parametric estimator of the cumulative hazards of infection with or without febrile disease plus cough in each intervention group [24]. We constructed a competing risks survival analysis model that accounted for the alternative modes of transmission and used it to infer the relative importance of alternative modes of transmission assuming that the risk of fever plus cough higher in aerosol transmission, compared with the other two modes. We assumed independent hazards over time of influenza transmission in households with one or more secondary cases. The cause-specific probability of aerosol transmission was estimated to measure the relative contribution of aerosol transmission among all three modes.

A mixture model was used to allow for a certain proportion (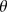) of subjects to be immune or not exposed, with the density of infection described as 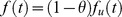, where 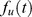 is the probability density function for the exposed and susceptible group. The time to infection (*T*) for each of three modes of transmission was assumed to follow a Weibull distribution with an identical shared shape parameter (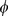) and mode-specific scale parameters (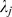). The sub-hazards for modes of transmission, *j* = 1, 2 and 3 representing contact, large droplets and aerosols respectively were written as follows:
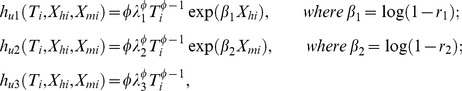
where 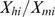 are the dichotomous indicator variables representing the allocation of hand hygiene/surgical mask interventions respectively to individual *i*, and 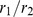 represent the relative risk reductions in contact/large droplet transmission by hand hygiene/surgical masks respectively. We assumed that the risk of fever plus cough caused by infections follows a Bernoulli distribution with mean parameter 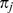, *j* = 1, 2, 3 for three arms, respectively. We estimated 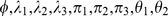. We were unable to estimate 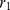 and 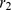 so we examined the estimates of the other parameters for a range of values of 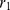 and 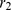. Further technical details of the model are provided in an earlier publication [9].

We performed statistical inference under a Bayesian framework, using Markov chain Monte Carlo (MCMC) to obtain parameter estimates from the posterior distributions [25]. We specified flat priors for each parameter. For each MCMC chain we ran 120,000 iterations, discarding the first 20,000 iterations as burn-in, and drawing every tenth subsequent value to compose the posterior distribution. All the statistical analyses were conducted in R version 2.15.1 (R Foundation for Statistical Computing, Vienna, Austria).

## Results

**Figure 1:**
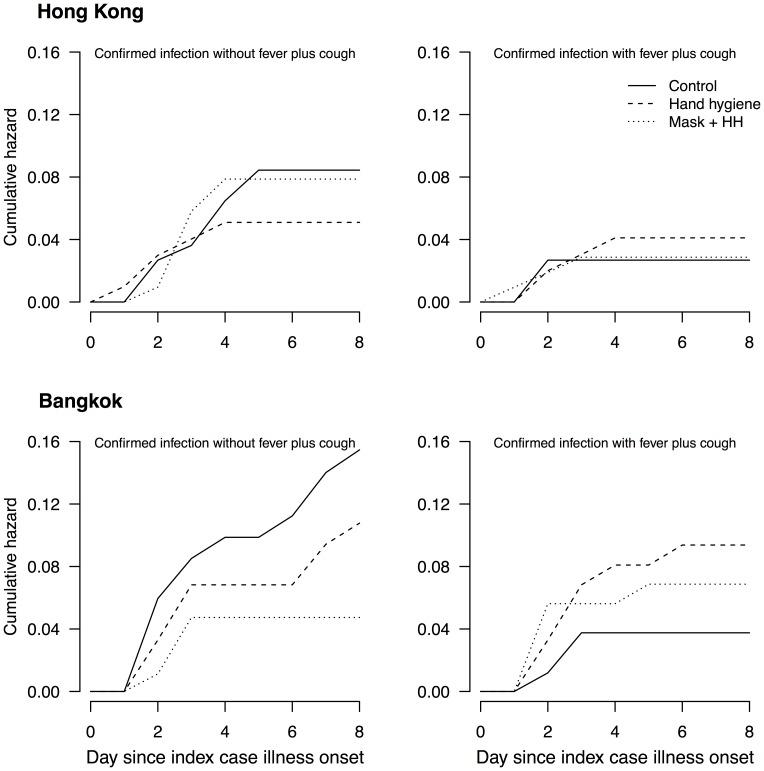
Cumulative hazards of RT-PCR-confirmed influenza B virus infections presenting with fever plus cough or not presenting with fever plus cough, among the household contacts in 104 and 113 households of index cases with RT-PCR-confirmed influenza B virus infection in Hong Kong and Bangkok, respectively.

In Hong Kong and Bangkok there were 104 and 113 households, respectively, with an index case with RT-PCR-confirmed influenza B virus infection. The characteristics of index cases and their household contacts are shown in Tables 2 and 3 for Hong Kong and Bangkok respectively. We examined the cumulative hazard of RT-PCR-confirmed influenza B virus infections for household contacts, and found increases in the risk of infection with fever plus cough, and decreases in the risk of infection without fever plus cough, in the intervention arms compared to the control arm. The change was particularly apparent in the households in Bangkok (Figure 1). To be more specific, we found a statistically significant decrease in the risk of infection without fever plus cough, in the hand hygiene plus face masks arm compared to the control arm in the households in Bangkok.

**Table 2:** Characteristics of index cases with confirmed influenza B virus infection and their household contacts in Hong Kong, by intervention group.

	Control		Hand hygiene		Face mask+hand hygiene	
Characteristics	n	(%)	n	(%)	n	(%)
*Index cases*	*35*		*36*		*33*	
Age group						
≤5 y	5	(14%)	3	(8%)	4	(12%)
6–15 y	25	(71%)	21	(58%)	21	(64%)
>16 y	5	(14%)	12	(33%)	8	(24%)
Male	16	(46%)	19	(53%)	10	(30%)
Median household size (IQR)	4	(3, 5)	4	(3, 4)	4	(3, 5)
*Household contacts*	*112*		*101*		*106*	
Age group						
≤5 y	6	(5%)	1	(1%)	5	(5%)
6–15 y	13	(12%)	12	(12%)	12	(11%)
16–30 y	21	(19%)	17	(17%)	17	(16%)
31–50 y	58	(52%)	48	(48%)	51	(48%)
>50 y	14	(12%)	23	(23%)	21	(20%)
Male	39	(35%)	40	(40%)	46	(43%)
Received seasonal influenza vaccination in the previous 12 m	15	(13%)	12	(12%)	14	(13%)

**Table 3:** Characteristics of index cases with confirmed influenza B virus infection and their household contacts in Bangkok, by intervention group.

	Control		Hand hygiene		Face mask+hand hygiene	
Characteristics	n	(%)	n	(%)	n	(%)
*Index cases*	*37*		*38*		*38*	
Age group						
≤5 y	12	(32%)	14	(37%)	10	(26%)
6–15 y	25	(68%)	24	(63%)	28	(74%)
>16 y	0	(0%)	0	(0%)	0	(0%)
Male	24	(65%)	23	(61%)	23	(61%)
Median household size (IQR)	2	(2, 3)	3	(2, 3)	3	(2, 5)
*Household contacts*	*84*		*91*		*89*	
Age group						
≤5 y	1	(1%)	5	(5%)	4	(4%)
6–15 y	10	(12%)	14	(15%)	10	(11%)
16–30 y	13	(15%)	18	(20%)	15	(17%)
31–50 y	49	(58%)	41	(45%)	37	(42%)
>50 y	11	(13%)	13	(14%)	23	(26%)
Male	35	(42%)	39	(43%)	33	(37%)
Received seasonal influenza vaccination in the previous 12 m	0	(0%)	0	(0%)	0	(0%)

**Figure 2:**
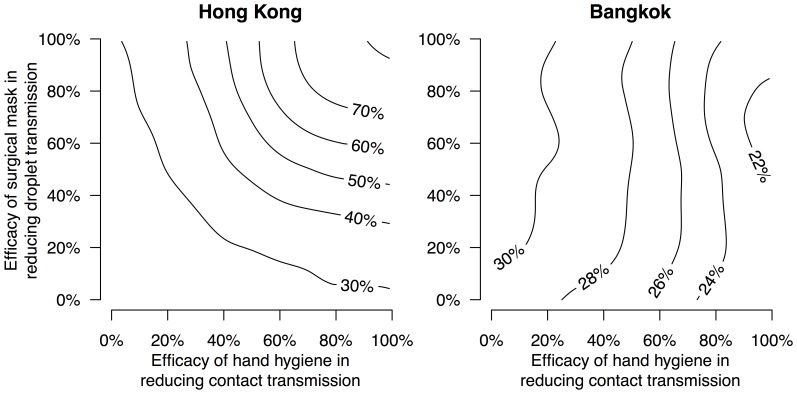
The relative importance (cause-specific probability) of aerosol transmission in households in Hong Kong and Bangkok. The contour lines show the proportion of secondary influenza B virus infections attributed to aerosol transmission in the control arm of each study, under varying assumptions about the efficacy of randomization to the hand hygiene and surgical mask interventions in reducing contact (x-axis) and droplet (y-axis) transmission respectively.

Under the scenario where randomization to the hand hygiene intervention reduced contact transmission by 50% while randomization to face mask and hand hygiene interventions reduced both contact and droplet transmission by 50%, we fitted the transmission model to the Hong Kong and Bangkok data. We estimated that in the absence of interventions, aerosol transmission was responsible for 37% and 26% of secondary infections in Hong Kong and Bangkok, respectively (Table 4). We also varied the assumed efficacy of hand hygiene and face masks from 0% to 100% and estimated the relative importance of aerosol transmission in the absence of interventions, which ranged from approximately 20% to 80% in Hong Kong and 20% to 32% in Bangkok (Figure 2).

**Table 4:** Point estimates and 95% credible intervals of model parameters under an exemplar plausible scenario that hand hygiene and surgical face masks reduced contact and droplet transmission respectively by 50% from the time of application of those interventions.

	Hong Kong(104 householdswith 319 contacts)		Bangkok(113 householdswith 264 contacts)	
Parameters	Estimate	(95% CI)	Estimate	(95% CI)
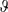 Shape of the Weibull distribution	2.16	(1.30, 3.12)	0.77	(0.39, 1.28)
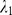 Force of contact transmission*	0.18	(0.01, 0.40)	0.16	(0.01, 0.48)
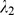 Force of droplet transmission*	0.20	(0.01, 0.40)	0.07	(0.00, 0.24)
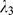 Force of aerosol transmission*	0.22	(0.02, 0.38)	0.08	(0.00, 0.25)
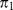 Risk of fever plus cough forinfections by contact route	23%	(1%, 66%)	25%	(1%, 63%)
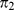 Risk of fever plus cough forinfections by droplet route	24%	(1%, 60%)	31%	(2%, 75%)
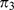 Risk of fever plus cough forinfections by aerosol route	56%	(26%, 97%)	72%	(41%, 99%)
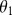 Proportion of household adultsimmune or not exposed	90%	(85%, 94%)	65%	(45%, 79%)
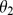 Proportion of householdchildren immune or not exposed	69%	(54%, 82%)	61%	(34%, 82%)

*The forces of infection in combination with a shared shape parameter determine the hazard associated with each competing mode of transmission. The relative contribution of each mode *j* is calculated as the cause-specific probabilities 
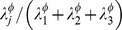
_._

**Figure 3:**
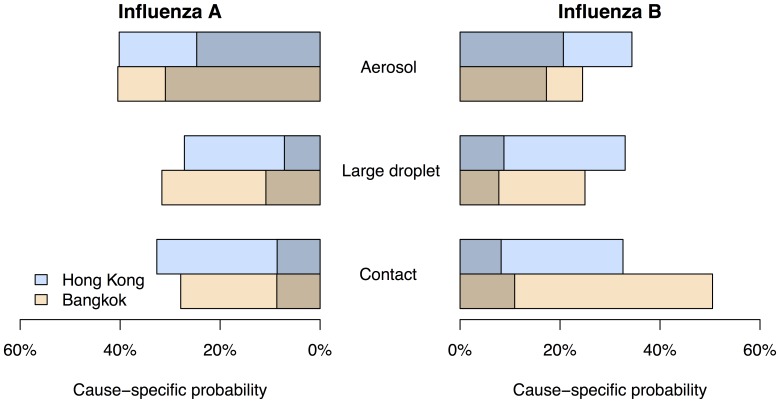
The proportion of all influenza A and B virus infections attributed to each mode in the control arms of the studies in Hong Kong (blue) and Bangkok (brown), and the infections associated with fever plus cough (darker shade) or not associated with fever plus cough (lighter shade). Data shown on influenza A were extracted from a previous study [9]. The contributions of the three modes sum to 100% within each geographic location and influenza type.

We compared the cause-specific probabilities of each mode of transmission as well as the associated illnesses in the control arm for influenza A and B virus infections, in Hong Kong and Bangkok respectively (Figure 3). Data for influenza A were extracted from a previous report [9]. Both influenza A and B virus infections attributed to aerosol transmission were associated with a higher risk of fever plus cough, compared with the other two modes of transmission. The point estimates of aerosol transmission were lower for influenza B compared to influenza A in both Hong Kong and Bangkok.

## Discussion

We propose that the mode of spread associated with an influenza B virus infection affects the probability of experiencing fever plus cough for that infection. Based on that hypothesis, we estimated that approximately 37% and 26% of transmission was via the aerosol mode in households in Hong Kong and Bangkok, respectively. However, we should exercise caution in interpreting these findings because we have not been able to find literature supporting the anisotropic nature of influenza B virus infection, whereas we previously described literature supporting this property for influenza A virus infections [9]. Nevertheless, patterns in secondary infections and disease in the controlled trials in Hong Kong and Bangkok were consistent with this hypothesis (Figure 1). This also implicitly suggested that though hand hygiene and face masks could reduce the risk of transmission through contact or large droplets, but meanwhile increase the risk of aerosol transmission, which was associated with a greater risk of illness with fever plus cough.

Whereas we previously estimated that approximately half of within-household transmission of influenza A virus could be associated with aerosols [9], here we estimated a slightly reduced importance of aerosols for influenza B virus (Figure 3). One explanation for such a difference could be the age mix of cases of influenza A versus B, if aerosol transmission were more important among adults than children. We did not have sufficient sample size in the present study to examine whether modes of transmission might vary by age, but this would be an interesting area for further exploration.

If aerosol transmission is indeed an important mode of spread of influenza B virus, this may have important implications for control efforts. In particular measures targeting contact transmission, such as hand hygiene, and measures targeting large respiratory droplet transmission, such as surgical face masks, may not be sufficient to substantially reduce the risk of transmission. Control measures that might reduce aerosol transmission indoors include improvement in ventilation [26], modification of humidity [27], or the use of personal protective equipment that is more effective against aerosols than surgical masks. While the use of N95 respirators may not be practical in community settings and fit-testing is unlikely although required for optimal performance, other types of face masks with improved filtration compared to standard surgical masks or procedure masks may be available in the future.

There are a number of limitations to our analysis. First, our model did not include the possibility of variability in infectiousness between index cases, variability in immunity to different modes of transmission, or variability in within-household transmissibility associated with physical dimensions of the home, ventilation rates etc, and inclusion of these or other factors potentially affecting transmission dynamics could be natural extensions to our model. Because interventions were allocated randomly among households, the possibility of confounding should be minimized. Second, our model implicitly assumes that only the first infectious exposure is relevant to susceptible contacts, and once infected by that first exposure, further exposures are unimportant. Our model could be modified to allow for multiple simultaneous exposures by one or more modes, if it were understood how this might affect the course of disease. Third, while we assumed that all infections of household contacts during the 7-day follow-up were acquired within the household, it is possible that some infections were acquired outside. However in a separate study with a similar design in Hong Kong we used molecular epidemiology analyses of virus sequence data to demonstrate that most secondary influenza cases acquired infection from within the household [28], and a similar observation was reported in a household transmission study in Canada [29]. Fourth, it is possible that some secondary influenza virus infections were not confirmed due to poor quality specimens collected during home visits, or if peak influenza B viral shedding in the respiratory tract occurred between home visits at 3-day intervals. We did include serological data although this could have provided additional information on infections among household contacts. Fifth, by recruiting in outpatient clinics and using a rapid test to screen index cases, we may have introduced selection bias towards index cases with more serious illness or higher levels of virus shedding, affecting the relative importance of different modes of transmission. Finally, we did not explicitly account for imperfect adherence to the interventions, although the parameters in our model account for moderate efficacy of interventions against specific modes of transmission. Further improvements in the model might be obtained by incorporating limited data on adherence that was mainly self-reported by participants.

In conclusion, we propose that the aerosol route may be an important mode of transmission of influenza B virus in households. Further studies of non-pharmaceutical interventions in households would be improved by more careful monitoring of viral contamination on surfaces [30], [31] and in the air, and inclusion of this information in transmission models.
